# Supplementary material for: Demographic causes of adult sex ratio variation and their consequences for parental cooperation
Source: Nat Commun. 2018 Apr 25;9:1651. doi: 10.1038/s41467-018-03833-5 (PMC5917032; doi:10.1038/s41467-018-03833-5)
Supplement: Supplementary file 1 — Supplementary Information [file 41467_2018_3833_MOESM1_ESM.pdf]

Demographic causes of adult sex ratio variation and their consequences for parental cooperation

Eberhart-Phillips et al.

Supplementary Information

Supplementary Table 1. Study site metadata: geographic location (see Fig. 1) and duration of monitoring effort.

Species	Population	Latitude	Longitude	Years monitored
C. nivosus	Mexico	23°54’N	106°57’W	2006-2012
C. alexandrinus	Turkey	36°43’N	35°03’E	1996-2001
	Cape Verde	15°8’N	23°13’W	2007-2015
C. thoracicus	Madagascar	22°6’S	43°15’E	2009-2015
C. marginatus	Madagascar			2009-2015
C. pecuarius	Madagascar			2009-2015
				43 years

Supplementary Table 2. Summary of parental care sex roles. Percentages reflect the within-population proportion of families with a given parental care system.

Species	Population	Bi-parental [95% CI]	Female-only care [95% CI]	Male-only care [95% CI]	*n*
C. nivosus	Mexico	9% [5, 14]	1% [0, 6]	90% [85, 94]	125
C. alexandrinus	Turkey	50% [40, 62]	9% [0, 21]	41% [31, 53]	78
	Cape Verde	71% [64, 77]	15% [9, 22]	14% [8, 21]	170
C. thoracicus	Madagascar	93% [86, 100]	0% [0, 12]	7% [0, 36]	14
C. marginatus	Madagascar	96% [91, 100]	4% [0, 12]	0% [0, 7]	23
C. pecuarius	Madagascar	0% [0, 10]	20% [11, 30]	80% [72, 91]	61
					471

Supplementary Table 3. Summary of hatching sex ratio data, where ρ is the average hatching sex ratio (expressed as the proportion of hatchlings in a brood that are male) and 95% CIs are calculated using a binomial distribution.

**Species**	**Population**	N_{Families}	$N_{\text{Hatchlings}}$	ρ	**95% CI**
C. nivosus	Mexico	198	484	0.469	[0.425, 0.514]
C. alexandrinus	Turkey	102	262	0.508	[0.447, 0.568]
	Cape Verde	107	197	0.477	[0.408, 0.547]
C. thoracicus	Madagascar	11	22	0.636	[0.423, 0.807]
C. marginatus	Madagascar	13	30	0.600	[0.419, 0.757]
C. pecuarius	Madagascar	72	144	0.528	[0.446, 0.757]

Supplementary Table 4. Sample size and over-dispersion summary of mark-recapture dataset used to estimate apparent survival.

Species	Population	Juveniles‡		Adults†		Total individuals	Median $\hat{c}$
		♀	♂	♀	♂		
C. nivosus	Mexico	438	388	221	212	1358	1.70
C. alexandrinus	Turkey	310	293	557	504	1664	1.49
	Cape Verde	377	383	254	213	1227	1.37
C. thoracicus	Madagascar	38	56	83	68	245	2.72
C. marginatus	Madagascar	76	96	99	95	366	1.31
C. pecuarius	Madagascar	274	286	382	416	1358	1.77
						6119	

‡Individuals first marked as hatchlings (i.e. known age).

†Individuals first marked as breeding adults (i.e. 1+ years old).

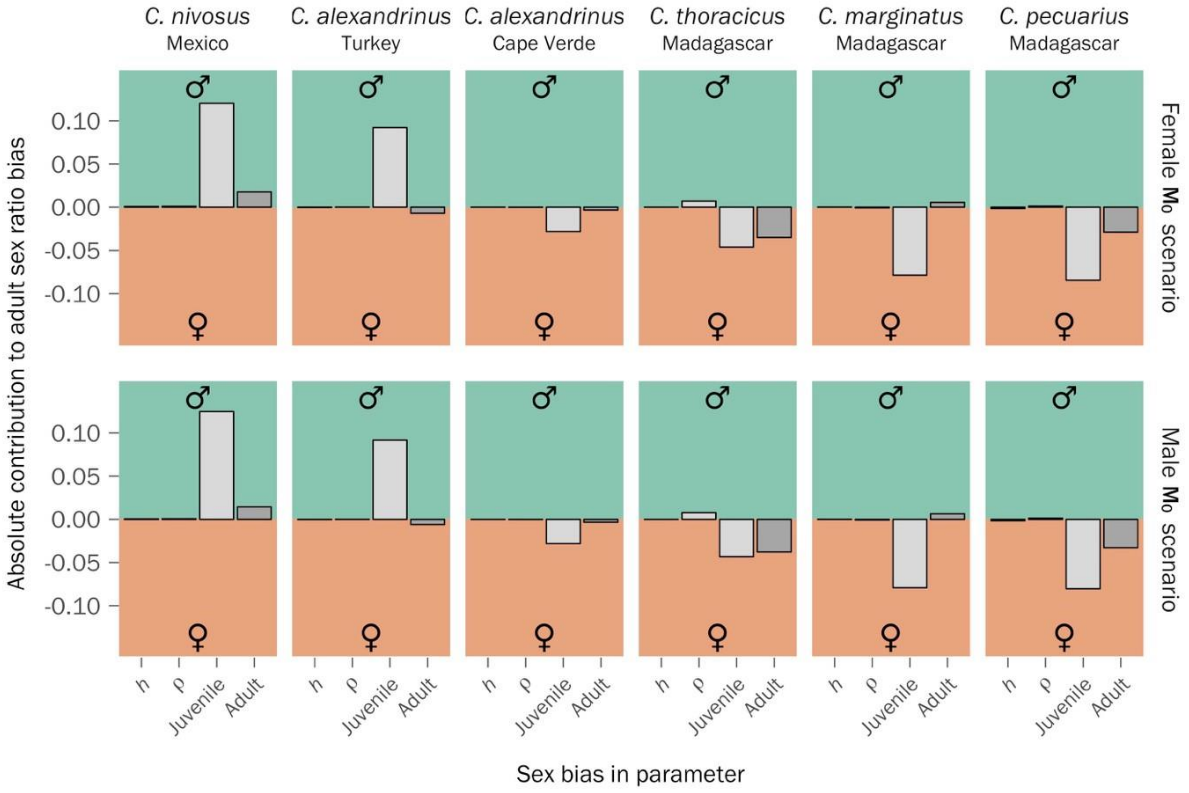

Supplementary Figure 1. Contributions of sex-specific parameters to adult sex ratio bias. These results are based on a life-table response experiment (LTRE) that compared the empirically-derived sex-specific model to null scenarios with no sex differences in demographic rates (top panel: $\mathbf{M_0}$ consists of female rates, bottom panel: $\mathbf{M_0}$ consists of male rates; Eq. 8) and a monogamous mating system (i.e. h = 1). Because ASR is measured as the proportion of the adult population that is male, LTRE statistics are negative for demographic rates that are female-biased in each population. Notation: h = mating system index (Eq. 6), ρ = hatching sex ratio, Juvenile = sex-biased apparent survival of juveniles, Adult = sex-biased apparent survival of adults.

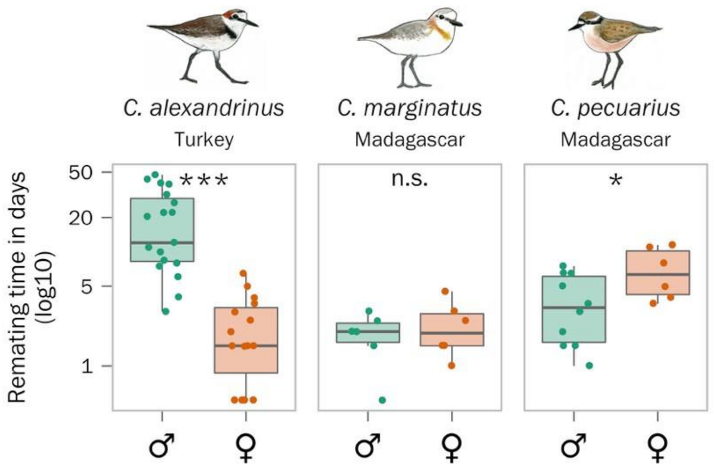

Supplementary Figure 2. Interspecific variation in sex-specific mating opportunities among three plover species. Experimental assessment of sex-differences in remating times in three of the six populations analysed indicate that *C. alexandrinus* males in Tuzla, Turkey (n = 19) take longer to find a mate than females (n = 15) after induced divorce. This trend is reversed in *C. pecuarius* ($n_{♂}$ = 10, $n_{♀}$ = 6) whereas there are no differences in the *C. marginatus* ($n_{♂}$ = 6, $n_{♀}$ = 6). Significant sex-differences are indicated by asterisks (***: P < 0.001, *: P < 0.05, n.s.: P > 0.05). Figure adapted from Parra et al.[1]. Original plover illustrations by L.J.E-P.

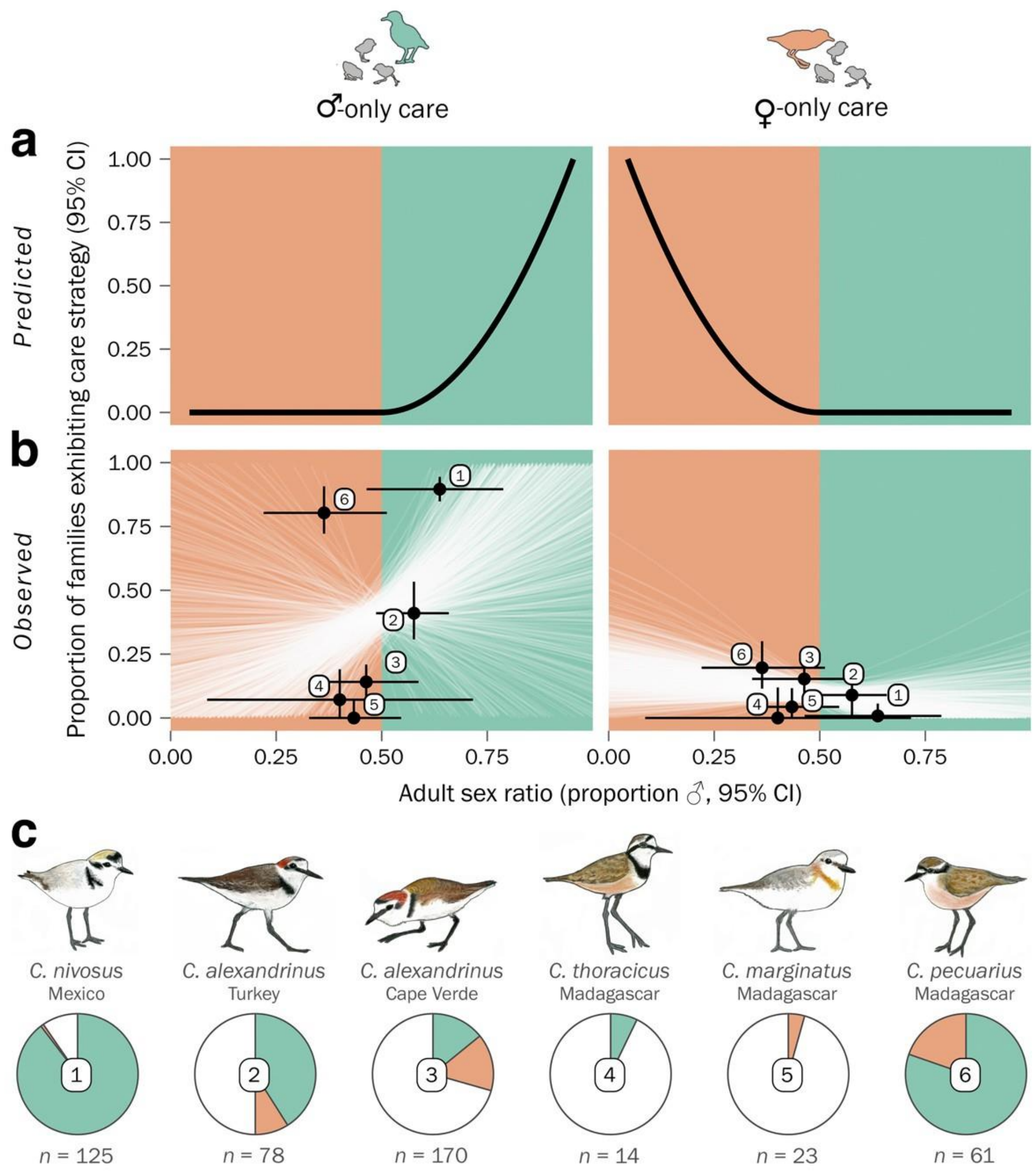

Supplementary Figure 3. Relationship between uni-parental care and the adult sex ratio. (**a**) Predicted prevalence of male-only care (left panel) or female-only care (right panel) in response to adult sex ratio variation. (**b**) Observed relationship between parental care strategies and adult sex ratio estimates among the six studied populations. Faint white lines illustrate each iteration of the bootstrap, which randomly sampled an adult sex ratio and parental care estimate from each population's uncertainty distribution and fitted them to the *a priori* exponential model (Eq. 11). (**c**) Proportion of monitored plover families that exhibit parental cooperation (white) or uni-parental care by males (green) or females (orange).

Sample sizes reflect the number of families monitored per population, circled numbers correspond to the data point labels shown in panel (b). Original plover illustrations and silhouettes by L.J.E-P.

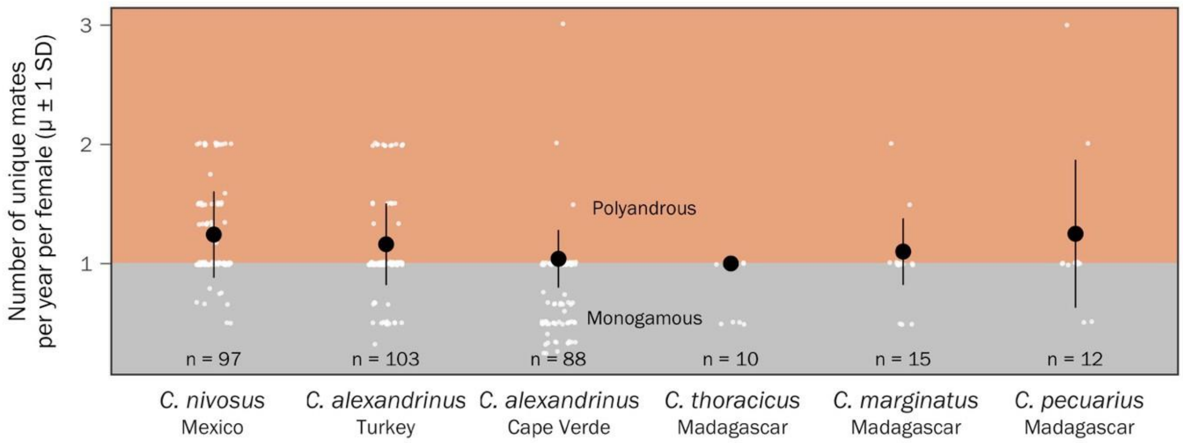

Supplementary Figure 4. Variation in annual female mating rates (μ) among the six plover populations. Sample sizes indicate the number of individual females in each population that had at least two recorded breeding attempts with identified male(s) during the study. Values below one represent females that bred over multiple years with the same mate (i.e. long-term monogamy), whereas values greater that one represent females that have had more than one mate per year (i.e. within season polyandry). Values equal to one represent individuals that have had one mate per year, but have switched mates between years (i.e. between season polyandry but within season monogamy). White data points illustrate individual females' mates per year (i.e. m_i / b_i in Eq. 5), and black points are population averages corrected for long-term monogamy according to Eq. 5 (μ ± 1 SD).

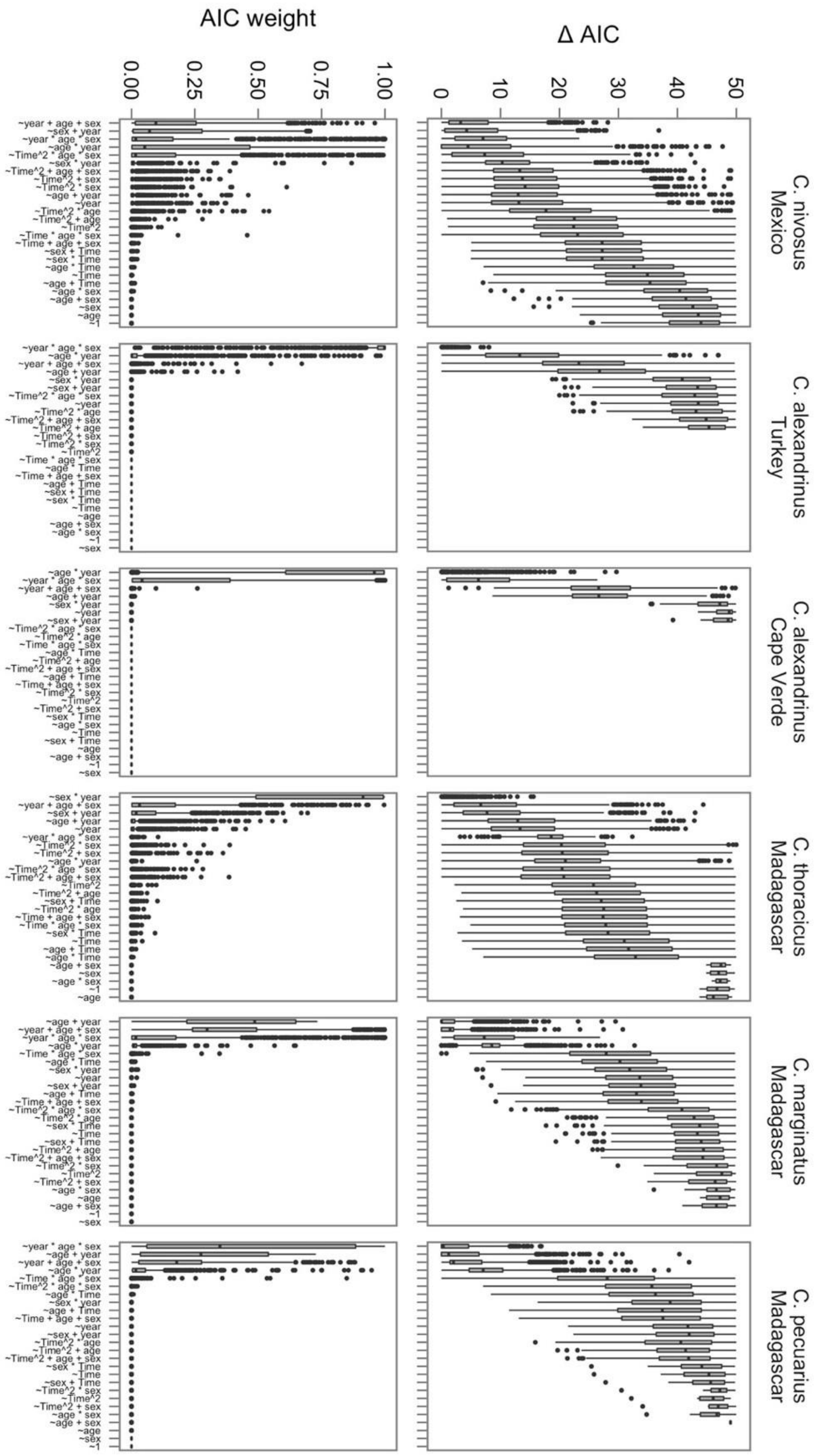

Supplementary Figure 5. Summary statistics of bootstrapped mark-recapture modelling of juvenile and adult encounter probability. Left panels illustrate variation in AIC_C w_i. Right panels illustrate variation in ΔAIC_C. Model structure of encounter probability (p) is shown as labels on the y-axes. See Methods for further details.

Supplementary References

1. Parra, J. E., Beltrán, M., Zefania, S., Remedios, dos, N. & Székely, T. Experimental assessment of mating opportunities in three shorebird species. *Anim. Behav.* **90**, 83–90 (2014).
