# Supplementary material for: Demographic causes of adult sex ratio variation and their consequences for parental cooperation
Source: Nat Commun. 2018 Apr 25;9:1651. doi: 10.1038/s41467-018-03833-5 (PMC5917032; doi:10.1038/s41467-018-03833-5)
Supplement: Supplementary file 3 — Description of Additional Supplementary Files [file 41467_2018_3833_MOESM3_ESM.pdf]

Description of Additional Supplementary Files

Supplementary Movie 1. **Field methods for capturing and monitoring plovers.** A movie illustrating our field methods for studying plovers in the wild.

Supplementary Data 1. .Rmd file needed to reproduce all our modelling and statistical analysis in R.
